# An Adversarial Learning Approach for Super-Resolution Enhancement Based on AgCl@Ag Nanoparticles in Scanning Electron Microscopy Images

**DOI:** 10.3390/nano11123305

**Published:** 2021-12-06

**Authors:** Li Fan, Zelin Wang, Yuxiang Lu, Jianguang Zhou

**Affiliations:** Research Center for Analytical Instrumentation, State Key Laboratory of Industrial Control Technology, Institute of Cyber-Systems and Control, Zhejiang University, Hangzhou 310027, China; 11732041@zju.edu.cn (L.F.); 12032052@zju.edu.cn (Z.W.); yuxianglu@zju.edu.cn (Y.L.)

**Keywords:** super-resolution, scanning electron microscopy (SEM), adversarial learning, AgCl@Ag microstructure

## Abstract

Scanning electron microscopy (SEM) plays a crucial role in the characterization of nanoparticles. Unfortunately, due to the limited resolution, existing imaging techniques are insufficient to display all detailed characteristics at the nanoscale. Hardware-oriented techniques are troubled with costs and material properties. Computational approaches often prefer blurry results or produce a less meaningful high-frequency noise. Therefore, we present a staged loss-driven neural networks model architecture to transform low-resolution SEM images into super-resolved ones. Our approach consists of two stages: first, residual channel attention network (RCAN) with mean absolute error (MAE) loss was used to get a better peak signal-to-noise ratio (PSNR). Then, discriminators with adversarial losses were activated to reconstruct high-frequency texture features. The quantitative and qualitative evaluation results indicate that compared with other advanced approaches, our model achieves satisfactory results. The experiment in AgCl@Ag for photocatalytic degradation confirms that our proposed method can bring realistic high-frequency structural detailed information rather than meaningless noise. With this approach, high-resolution SEM images can be acquired immediately without sample damage. Moreover, it provides an enhanced characterization method for further directing the preparation of nanoparticles.

## 1. Introduction

In recent years, researchers have developed many kinds of new nanomaterials with unique structures and properties [1,2,3,4]. These nanoparticles have motivated tremendous applications in many fields, such as drug delivery, detection, and optics [5,6,7,8]. On top of that, the usage of nanoparticle catalysts in the broad field of catalysis has been more attractive [9,10,11]. In particular, the research has demonstrated that the smaller the size of metal nanoparticles, the higher surface area/volume ratio they had, and therefore, the higher catalytic activity they can obtain. Nanoparticles tend to have greater catalytic activity when they are smaller than 10 nm, until monoatomic particles are prepared, their atomic efficiency will be maximal [12]. However, as the size of metal nanoparticles substantially shrinks, it is more difficult to characterize them.

Analytical tools for characterizing the structure of nanomaterials include X-ray Photoelectron Spectroscopy (XPS), X-ray Diffraction (XRD), Transmission Electron Microscopy (TEM), and Scanning Electron Microscopy (SEM). Among them, SEM is the most versatile and commonly used in this field. By launching a focused electron beam at a specimen, and detecting the electron emission of the sample, SEM achieves high spatial resolution images containing topographic and compositional information of the surface [13]. It also has fast acquisition and large magnification, which make it convenient for real-time high-sensitivity digital processing [14]. Nonetheless, with the smaller size of nanoparticles, the existing resolution of SEM cannot keep up with the characterization requirements. Consequently, increasing the resolution and characterization capabilities of SEM is highly desirable.

Generally speaking, there are two ways to optimizing the resolution of SEM imaging systems: (1) hardware methods and (2) computational methods. First, some ultra-precision hardware components can improve the resolution performance to a certain extent [15]. Whereas most of these hardware components are high priced and lead to increases in costs. More to the point, the focused electron beam launched by the SEM can destroy the initial structure of nanomaterials, especially for some electron beam-sensitive materials [16,17]. For example, AgCl nanoparticles will be reduced to Ag nanoparticles under the electron beam. Based on the above, if nanoparticles are required to be imaged at higher resolution, greater speed without destroying, and using computational methods for resolution improvement of SEM images will be more suitable.

In order to improve SEM image resolution, many computational approaches were presented. These methods can be divided into the following categories: (1) Model-based super-resolution (SR) [18,19]: These methods model the degradation process of images based on a prior model and regularize the reconstruction images according to the features of the projections. However, these algorithms can obtain an ideal image quality only on the premise that the model-based priors are valid; (2) Learning-based super-resolution [20,21,22]: These methods can be trained with a dataset made up of low-resolution (LR) and high-resolution (HR) images pairs to learn a nonlinear mapping, then restore the missing high-frequency information and greatly enhance the image quality. Moreover, as long as the model is trained, SR images can be achieved simply through feed-forward propagation, which saves a lot of time and decreases the computational expense. These methods have exhibited great potential in the SEM imaging field.

However, there still exist some main problems with the application of learning-based SR approaches in SEM imaging. First, due to the existence of the residual network, the SR networks are able to construct deeper. However, simply stacking residual blocks can hardly obtain better improvements. In this condition, the detailed components of an image are often likely to become smooth in the SR output. Second, most SR approaches commit to minimizing pixel-wise mean squared error (MSE) between the ground truth image and the super-resolved image. Because minimizing pixel-wise errors can be regarded as maximizing peak signal-to-noise ratio (PSNR) [23], thus higher PSNR leads to image blurring and lack of high-frequency details rather than a perceptually better image. Third, while generative adversarial network (GAN) [24] based methods make great progress compared to previous approaches in the aspect of perceptual quality, they are more likely to generate high-frequency noise or artifacts in the network outputs. Thus, the most protruding problem is to get the perceptually pleasing images and suppress noise at the same time.

Together with these technical problems described above, the goal of this work is to present an adversarial learning based method to enhance the resolution of the SEM imaging system. First, we determine to develop a staged loss-driven neural networks model architecture for SEM image super-resolution. By training this model with two different loss functions in two distinct stages, we demonstrate the feasibility of this model architecture on the SEM image super-resolution problem. According to this model, we analyze the performance of the model in both pixel and frequency domains and evaluate the model robustness and denoising performance by monitoring the values of PSNR and Structural Similarity (SSIM) [25] in the training process, then deliver the quantitative results compared to other popular approaches. In addition to these quantitative evaluations of image quality measures, we conduct a controlled catalytic experiment to further apply and prove the effectiveness of our proposed method.

## 2. Materials and Methods

### 2.1. Row Data

We use AgCl@Ag test specimens as the row data of our super-resolution task, which applied by our colleagues. These test specimens have random silver nanoparticles on the AgCl surface varying sizes ranging from 20–100 nm.

The studies were conducted using a SEM (3.0 kV, SU70, Hitachi, Tokyo, Japan). The images were acquired under the instrumental conditions of working distance: 11,200 μm, emission current: 35,000 nA, size of the condenser aperture: 50 μm, type of the secondary electron detector: Everhart Thornly Secondary Electron Detectors (upper and lower detectors). The image dataset that we use to train the model was composed of paired high- and low-resolution SEM images of our test specimen, every pair of images were taken from the same area of the specimens. The SEM image pairs for the network training can be obtained by first capturing the high-resolution images, and then reducing the magnification factor for 4-fold and taking a picture holding on the same field of view. In this condition, the resolution of the image can be restricted by the number of pixels, we can treat the lower-resolved SEM images as the distortion versions of the higher magnification images.

The SEM images that we applied for our network training, validation and testing were obtained from the raw data without any further image quality improvement process, such as smoothing, denoising, etc., just in case that additional processing causes the image distortion and some information will be lost.

### 2.2. Datasets

For the nanoparticles SEM images, 4000 pairs of images (1280 × 960 pixels) were separated into the training set (3940), validation set (20), and test set (40). Then we randomly cropped and rotated all the images into a total of 120,000 pairs of partial-overlapping patches (HR: 296 × 296 pixels, LR: 74 × 74 pixels) for data augmentation; 1200 pairs of these patches were removed from the datasets due to the severe blur and color deviation between the images. All the datasets were made in the same way, and each dataset was applied to the same network model.

### 2.3. Neural Networks

The network model employed in this work was an adversarial learning based model, this kind of architecture uses a generator network (G) to transform the low-resolution images into the super-resolution enhanced images, besides, a discriminator network (D) can help the generator network (G) to narrow the gap between realistic high-resolution images and the generated ones. In this research, a residual channel attention network (RCAN) [26] is used as the generator. RCAN refers to a type of very deep convolutional network which is composed of residual channel attention block (RCAB) and residual in residual (RIR) structure, it consists of a couple of residual groups with the addition of long skip connections (LSC) [27]. Each residual group includes several residual blocks coupled with short skip connections (SSC). The effect of RIR is to enable low-frequency information to pass through skip connections and use the main network to concentrate on learning high-frequency information. In addition, the channel attention mechanism [28] can readjust channel-wise characteristics by calculating the interdependence among channels. It strengthens the representation of the network by enhancing the quality of spatial encodings in the feature hierarchy.

**Figure 1:**
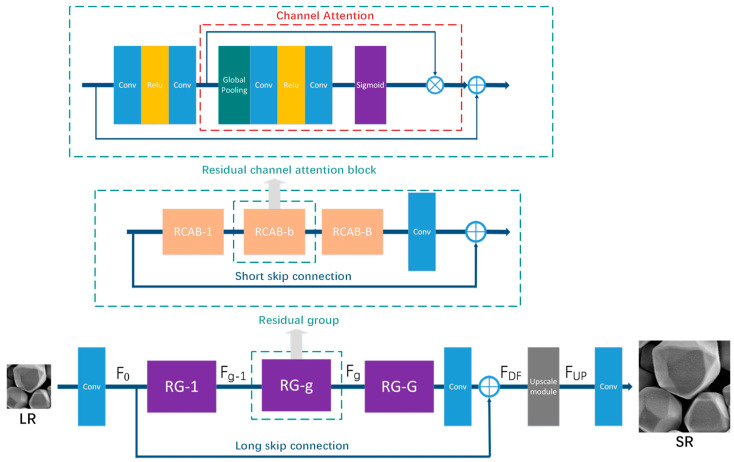
Architecture of the generator network which formed by residual in residual (RIR) structure. Channel attention (CA) mechanism integrated into residual block (RB) to form a residual channel attention block (RCAB). 20 RCAB with short skip connection (SSC) formed a residual group (RG). RIR structure consisted of 10 RG blocks and a long skip connection. This generator architecture can synthesis highly accurate SR images in the task.

As exhibited in Figure 1, Given $I_{LR}$ as a LR image input, using a convolutional layer to initially extract the shallow feature $F_{0}$ of the input:

(1) $$F_{0}=\mathrm{Conv}\left(I_{LR}\right)$$

$F_{0}$ is taken as the input of the RIR module for deep feature extraction as follows:

(2) $$F_{DF}=H_{RIR}\left(F_{0}\right)$$

where $H_{RIR}\left(\cdot \right)$ denotes a very deep RIR structure. The output was extracted as a deep feature, which can be upscaled through an upscale module [29]:

(3) $$\;F_{UP}=H_{UP}\left(F_{DF}\right)\;$$

where $H_{UP}\left(\cdot \right)$ and $F_{UP}$ represent an upscale module and upscaled feature separately. The sub-pixel convolution operation was used to aggregate low-resolution feature maps to help restore the HR image. This process is shown as:

(4) $$\;I_{SR}=H_{REC}\left(F_{UP}\right)=H_{RCAN}\left(I_{LR}\right)\;$$

where $H_{REC}\left(\cdot \right)$ and $H_{RCAN}\left(\cdot \right)$ define the reconstruction layer and the function of RCAN separately.

**Figure 2:**
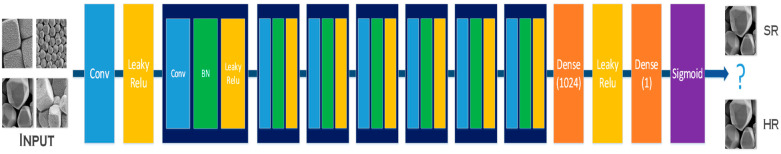
Architecture of our discriminator network that was used to distinguish generated SEM images from ground truth in both pixel and feature perspectives.

On the other hand, a VGG-based network [30] was used as the discriminator architecture which is shown in Figure 2. The discriminator differentiates generated images from input data in two perspectives: pixel and feature. The input of the image discriminator is the images in the pixel domain. Besides, the input of the feature discriminator is a feature map that was extracted by the middle layers in the network. The feature discriminator’s goal is to distinguish network output from ground truth images according to the intermediate feature map. As the feature map contains the encoded structural information, the feature discriminator can differentiate images based on structural components, so that the generator is more likely to generate authentic structural details rather than arbitrary noise.

### 2.4. Training

**Figure 3:**
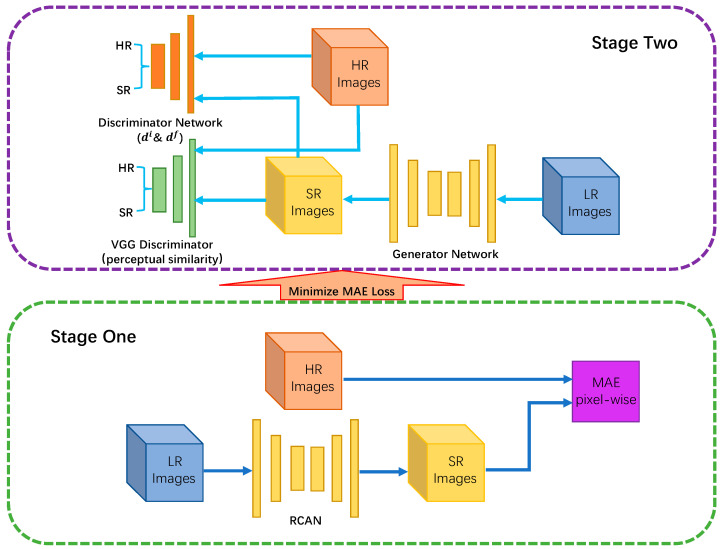
Data flow and system functional diagram of our approach. The dark blue line represents the data flow in stage one, as well as the light blue line represents the data flow in stage two. When MAE loss is minimized in stage one, it runs into stage two.

The training process of our approach can be divided into two stages. The data flow and system functional diagram can be visualized in Figure 3.

In stage one, we train the RCAN network by Giving a training set ${\left\{I_{LR}^{i},I_{HR}^{i}\right\}}_{i=1}^{N}$, which consists of N LR inputs and the corresponding HR image. The objective is to minimize mean absolute error (MAE) loss ($L_{1}$ loss function) [31]:

(5) $$L_{MAE}=\frac{1}{N}\sum_{i=1}^{N}{∥G_{RCAN}\left(I_{LR}\right)-I_{HR}∥}_{1}$$

The result that we obtained from stage one has already reached a satisfactory PSNR. However, it is still hard to synthesize perceptually pleasing output with ideal high-frequency detailed texture.

To optimize perceptual quality, we determined to import the GAN framework in stage two. The GAN framework was defined to solve a mini-max problem as:

(6) $$\begin{array}{c} \underset{G}{min}\underset{D}{max}E_{I_{HR}∼p_{\mathrm{train}\;}\left(I_{HR}\right)}\left[\log D\left(I_{HR}\right)\right]+\\ {\;E}_{I_{LR}∼p_{G}\left(I_{LR}\right)}[\log\left(1-D\left(G\left(I_{LR}\right)\right)\right] \end{array}$$

where $G\left(I_{LR}\right)$ is the generator super-resolution output of $I_{LR}$, D stands for the discriminator network which is trained to differentiate super-resolved images from the ground truth.

In stage two. We train the generator which was the same as the RCAN network in stage one with discriminators. Unlike the $L_{1}$ loss function in stage one, the total objective loss function of the generator is defined as:

(7) $$L_{gen}=L_{perc}+\lambda \left(L_{g}^{i}+L_{g}^{f}\right)$$

where $L_{perc}$ represents a perceptual similarity loss which enables SR outputs to look more similar to the ground truth (HR) images. $L_{g}^{i}$ stands for an image loss for the generator to generate high-frequency information in the pixel domain. $L_{g}^{f}$ represents a feature loss to encourage the generator to generate real structural information in the feature domain. λ stands for the loss terms weights. The discriminators $d^{i}$ and $d^{f}$ are trained by minimizing loss functions $L_{d}^{i}$ and $L_{d}^{f}$. In general, we optimize the whole network by alternately minimizing $L_{d}^{i}$, $L_{d}^{f}$ and $L_{gen}$.

The perceptual similarity loss $L_{perc}$ [32] between $I_{SR}$ and $I_{HR}$ is defined in the following way. First, $I_{SR}$ and $I_{SR}$ are feed into a pre-trained recognition network, then extract the m-th layer’s feature maps of the two images from the pre-trained network. The $L_{perc}$ is defined as MSE difference between the two feature maps:

(8) $$\;L_{perc}=\frac{1}{W_{m}H_{m}C_{m}}\sum_{i}^{W_{m}}\sum_{j}^{H_{m}}\sum_{k}^{C_{m}}{\left(\phi_{i,j,k}^{m}\left(I_{SR}\right)-\phi_{i,j,k}^{m}\left(I_{HR}\right)\right)}^{2}$$

where $W_{m}$, $H_{m}$, and $C_{m}$ denote the feature map $\phi^{m}$ dimensions of the m-th layer. VGG-19 was used as the recognition network. $\phi^{m}$ stands for the ReLU layer’s output before the m-th pooling.

The loss term $L_{g}^{i}$ which represent the image loss of the generator and the loss function $L_{d}^{i}$ which stands for the image discriminator are determined by:

(9) $$L_{g}^{i}=-\log\left(d^{i}\left(I_{SR}\right)\right)$$

(10) $$L_{d}^{i}=-\log\left(d^{i}\left(I_{HR}\right)\right)-\log\left(1-d^{i}\left(I_{SR}\right)\right)$$

where $d^{i}\left(I\right)$ is the output of the pixel domain discriminator $d^{i}$.

The loss term $L_{g}^{f}$ which represents the feature loss of the generator and the loss function $L_{d}^{f}$ which stands for the feature discriminator are determined by:

(11) $$L_{g}^{f}=-\log\left(d^{f}\left(\phi^{m}\left(I_{SR}\right)\right)\right)$$

(12) $$\;L_{d}^{f}=-\log\left(d^{f}\left(\phi^{m}\left(I_{HR}\right)\right)\right)-\log\left(1-d^{f}\left(\phi^{m}\left(I_{SR}\right)\right)\right)$$

where $d^{f}\left(\phi^{m}\right)$ is the output of the feature domain discriminator $d^{f}$. As features are equal to extracted image structures, our generator is stimulated to synthesize authentic high-frequency structural information rather than noise. Both the feature losses and the perceptual similarity loss relies on feature maps, the perceptual similarity loss improves perceptual consistency between $I_{SR}$ and $I_{HR}$, but the feature losses $L_{a}^{f}$ and $L_{d}^{f}$ allow synthesizing more visually desirable details in the image.

For both training stage one and stage two, we defined an intensity normalization of $I_{HR}$ and $I_{LR}$ to [−1, 1]. We used Adam optimizer and the hyper-parameter $\beta_{1}$ = 0.9. We defined the weight λ in Equation (7) as $10^{-3}$. Regarding $\phi^{m}$ in Equations (8), (11) and (12), we used the Conv5 layer of VGG-19 in the experiments as Conv5 often produces better outputs than other layers in many tasks. We rescaled VGG feature maps $\phi^{m}$ by a scale factor of 1/12.75 before we computed loss terms to balance different loss terms.

In stage one, we performed 15 epochs which included 7500 iterations for our randomly sampled training dataset. The initial learning rate was set for stage one as $10^{-4}$ and decreased it by 1/10 while the loss stopped descending. When the learning rate reached $10^{-6}$, we kept up the value without any change. In stage two, we also ran 15 epochs for adversarial training, which contained 7500 iterations per epoch. We set up the learning rate at $10^{-3}$ for the first five epochs, $10^{-4}$ for the next five epochs, and $10^{-5}$ for the last five epochs in stage two.

The networks were developed based on Keras with a TensorFlow 1.9.0 backend. In addition, training this task is supported by NVIDIA Quadro GV100 graphical processing unit (GPU) with 32GB of graphical memory.

## 3. Results

**Figure 4:**
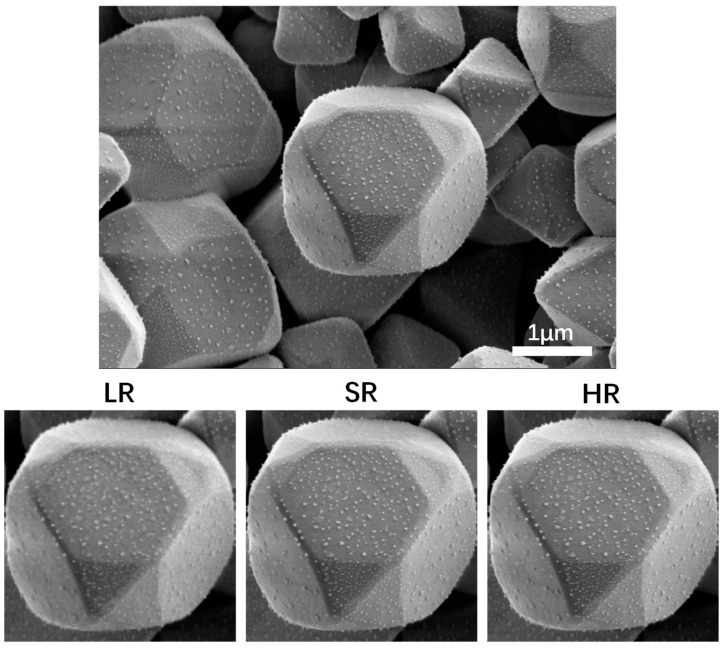
Test example of AgCl@Ag SEM image. The original SEM image with the cropped LR compared to the SR and HR versions.

This super-resolution approach enables us to increase the resolution of lower resolution SEM images 4-fold in a very short time, even so, the network’s SR output can still accurately match the high-resolution SEM images of the same region of interest. Taking an AgCl@Ag microstructure as a test specimen, the visualization results of the SEM images super-resolution can be directly seen in Figure 4. In the results section, we studied the resolution influence and application of our model. When comparing low-resolution input, stage one model output, stage two model output, and high-resolution ground truth image, we use the abbreviations LR, S1, S2, and HR to represent them respectively.

### 3.1. Spatial Frequency Analysis

**Figure 5:**
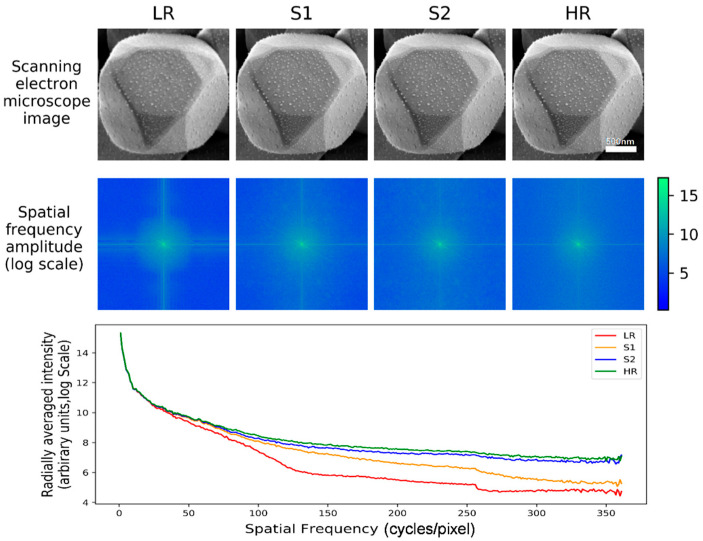
Spatial frequency analysis. Top: The AgCl@Ag example of the super-resolution network input images (LR) compared to stage one, stage two output images and the ground truth (HR) images. Middle: spatial frequency distributions of the four images on the top. Bottom: the plot of radially-averaged intensity of the above distributions.

An intuitive way to embody the performance of our method can be embodied in the spatial frequency analysis, the results are reported in Figure 5. This figure compares the spatial frequencies of the LR, S1, S2 and HR SEM images. From this comparative analysis, we can see that LR and S1 lines in radially averaged intensity plots had stronger attenuation with the increases of spatial frequency. That was because they lacked high-frequency signals. Whereas the S2 line is much better than LR and S1, especially in high-frequency details. Therefore, the network final output (S2) image’s spatial frequency distribution is identical to the ground truth (HR) image.

### 3.2. Pixel Precise Performance Analysis

**Figure 6:**
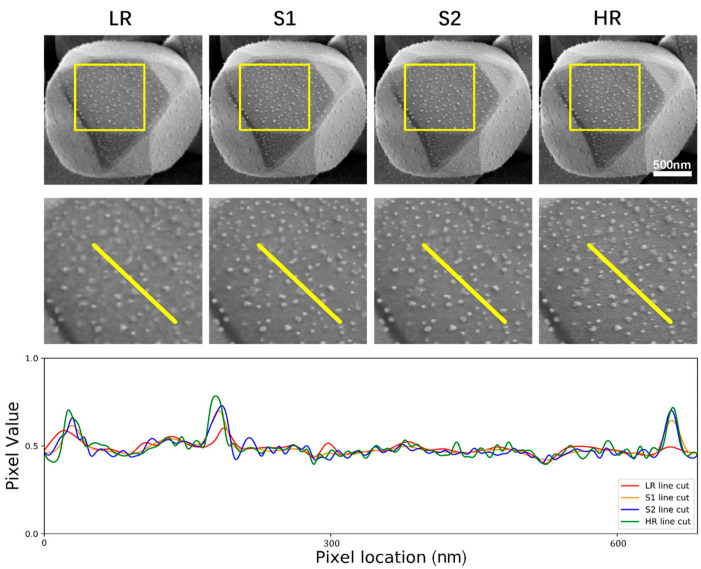
Visual results and pixel intensity. The top row displays the AgCl@Ag example of the network input images compared to the network output of each stage and the HR SEM images. The middle row displays the zoomed-in region framed in the top row images. The bottom row displays the pixel intensity values obtained from the yellow line cuts in the middle row images. The S2 line cut which is the final output of the network is almost reconstructed coincident with the HR line except for some acceptable noise.

A demonstration of the visual results and pixel precise performance is shown in Figure 6, which exhibits a random test example of nanoparticles that are unclear in the LR image but become clear and sharpened after the usage of our method. Pixel-intensity cross-sections are demonstrated to show the optimization process of resolution enhancement in each stage more clearly. From this example we can see that the pixel intensity value of the S1 line cut is a little smoother, it is more like the LR line cut. However, the S2 line cut is well approximated with high frequency. It depicts that our network is capable of gradually improving the spatial details that are not clear in the LR SEM images and matching the pixel values with the corresponding HR SEM images stage by stage. This can be evident in the exterior outline of the silver nanoparticles displayed in Figure 6.

### 3.3. Model Performance Quantitative Metrics

**Figure 7:**
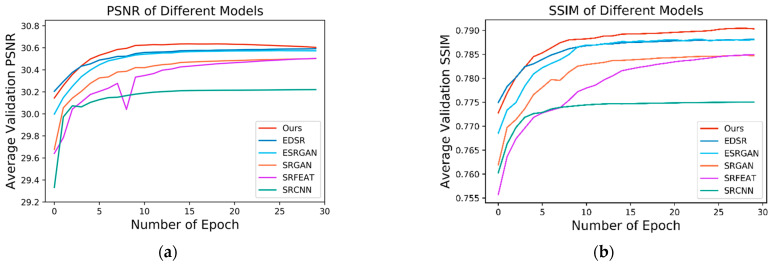
PSNR and SSIM analysis on SEM image super-resolution task in the training process. (**a**) Average PSNR monitoring results of compared models on validation set. (**b**) Average SSIM monitoring results of compared models on the validation set.

Here we monitored our network performance with other advanced approaches during the training process. Our approach is able to retain high-frequency textures to restore more authentic detailed components and suppress noise in a lower range simultaneously. In the first 15 epochs, our method is more like a PSNR-oriented method driven by the MAE loss in stage one. This stage mainly focuses on suppressing noise and artifacts. Nevertheless, judging by human observers, it is still restricted by some limitations. Since MSE or MAE loss assumes that the influence of noise has nothing to do with the local feature of the image. On the contrary, the sensitivity of the Human Visual System (HVS) to noise relies on local contrast, structure, and luminance [25]. In this term, we introduce the GAN-based model in stage two to improve structural identification. We introduce perceptual similarity loss, image loss and feature loss to lead our model to synthesize images with perceptually valid details in the last 15 epochs. Choosing PSNR and SSIM as the image quality evaluation metrics, we monitored the result values of the validation set in each epoch and compared our method with SRCNN [33], SRFEAT [34], SRGAN [35], ESRGAN [36] and EDSR [37] in Figure 7, our proposed approach obtains the best score in the SEM image super-resolution task. Based on the monitoring results, it indicates that our proposed method can converge stably and generate more visually pleasant results without severe blurriness or high-frequency artifacts than the competing methods.

We also quantitatively analyzed the super-resolution performance with the validation dataset and test dataset in terms of three image quality evaluation metrics that are broadly used: PSNR, SSIM, and Information Fidelity Criterion (IFC) [38]. The quantitative results demonstrate that our method not only obtains the highest scores in both PSNR and SSIM, which outperform other state-of-the-art methods. In addition, we added the IFC value which has a high correlation with the human perception of high-resolution pictures as another evaluation criteria. Table 1 shows the quantitative results proving that our approach could provide better visualization of microarchitecture in the SEM image resolution task which is basically identical to the monitoring results exhibited in Figure 7.

### 3.4. Effectiveness Verification

Although quantitative results have shown some advantages of the network, in practical applications, there is no reference (HR) image for us to verify it. To further evaluate the efficacy of our algorithm, we determined to use a controlled experiment to examine whether the high-frequency components were synthesized by the networks for real or not.

**Figure 8:**
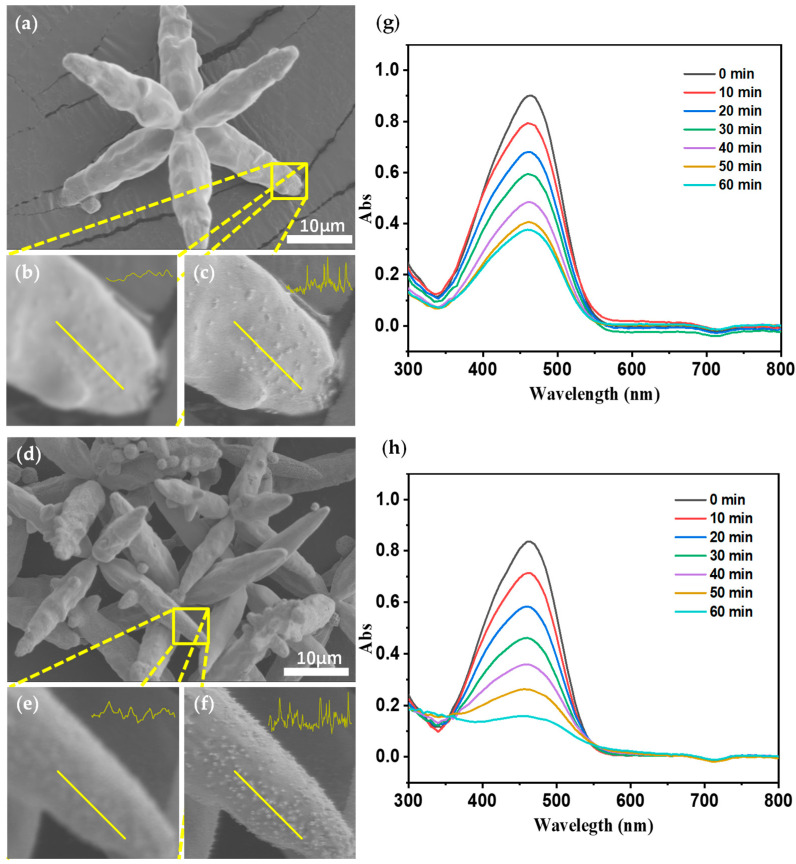
Original SEM image of AgCl@Ag particles with few Ag particles (**a**), zoomed in image of region of interest (ROI) (**b**), super-resolved image of ROI (**c**); original SEM image of AgCl@Ag particles with more Ag particles (**d**), zoomed in image of ROI (**e**), super-resolved image of ROI (**f**–**h**) UV-Vis spectra of MO under conditions of catalytic degradation of less and more Ag particles, respectively.

Based on the research of our colleagues [39,40], with the increase of Ag nanoparticles on an AgCl@Ag microstructure, its catalytic activity will become better. In this article, we prepared two AgCl@Ag particles with different Ag content by adjusting the molar ratio of NaBH_4_ to AgCl, labeled AgCl@Ag-1 and AgCl@Ag-2 respectively (see details in Supplementary Material Figures S1–S3). From the original SEM images of AgCl@Ag-1 (Figure 8a) and AgCl@Ag-2 (Figure 8d), it is difficult to see the Ag particles on the surface. Then we zoomed in on the region of interest (ROI) (Figure 8b, e), it is still hard to distinguish if there are Ag particles on the microstructure. To determine whether the Ag nanoparticles are resolvable, we use our method to process the ROI images. From the super-resolved images (Figure 8c,f) we can clearly see the Ag particles on the AgCl@Ag surface, and it is evident that AgCl@Ag-2 has more Ag particles than AgCl@Ag-1 which is shown from the pixel intensity of the yellow line cuts in the images. According to previous research results, AgCl@Ag-2 should have a better catalytic effect than AgCl@Ag-1.

To prove the super-resolved results above, we conducted a catalytic experiment. According to the Beer–Lambert Law, a decrease in MO concentration will lead to a decrease in the UV-Vis absorption spectrum of MO. In this case, degradation of MO by the photocatalytic activity of AgCl@Ag nanoparticles will result in a decrease in MO concentration. While keeping other reaction conditions consistent, use the same mass of AgCl@Ag-1 and AgCl@Ag-2 for photocatalytic degradation of methyl orange (MO) (λ > 420 nm). The real-time monitoring results of the UV-Vis spectroscopy of AgCl@Ag catalytic degradation of MO (Figure 8g,h) show that under the same reaction time (60 min), AgCl@Ag-2 degrades more MO than AgCl@Ag-1 (compared to AgCl particles displayed in Figure S4). It indicates that AgCl@Ag-2 shows a better catalytic effect than AgCl@Ag-1, which is highly consistent with the above super-resolved SEM images. These results confirmed the existence of silver particles and further verified that our network generated more realistic high-frequency information rather than various noise or artifacts.

To apply this method in the nanoparticles study, it can be trained to capture and synthesize more detailed features of nanoparticles. This procedure not only can realize large range high-resolution scanning of SEM but also request low computational and time costs. This approach can also be applied to other image processing tasks, such as segmentation or registration because high-resolution images generated by the super-resolution model provide more detailed information which can greatly enhance the performance in these tasks.

## 4. Conclusions

In this study, we established an adversarial learning-based networks training framework for SEM super-resolution imaging. Using paired data, our network learned complex structural characteristics more efficiently and achieved significant super-resolution gain through two stages and two different loss functions. In general, our proposed method shows robustness and high performance in producing promising results which restored detailed structural features and suppressed image noise. We also proposed an effectiveness verification method of super-resolution adapted to our specific task. In the controlled catalytic experiment, it confirmed that the super-resolution results obtained by this method tend to generate realistic high-frequency texture features in the SEM images, which are highly identical with the quantitative metrics evaluations according to three common image quality measures. By using this approach, we can easily visualize the nanoparticles, and then further direct the preparation of nanoparticles, such as through preliminary evaluation of the surface morphology of nano-catalytic materials, we can continue to optimize the prepared microstructures to meet specific catalytic performance.

## Figures and Tables

**Table 1:** Quantitative results of SEM image super-resolution in validation dataset and test dataset. Best results are highlighted.

Model	PSNR		SSIM		IFC	
	*Validation*	*Test*	*Validation*	*Test*	*Validation*	*Test*
Bicubic	28.8893	32.7277	0.7237	0.7986	2.2080	2.6611
SRCNN	30.1828	34.3070	0.7741	0.8308	2.8027	3.4853
SRFEAT	30.4520	34.5693	0.7844	0.8370	2.9933	3.7132
SRGAN	30.4613	34.6050	0.7844	0.8374	3.0142	3.7318
ESRGAN	30.5191	34.6965	0.7876	0.8395	3.0389	3.8046
EDSR	30.5335	34.6844	0.7880	0.8393	3.0543	3.8150
OURS	30.5446	34.7389	0.7903	0.8425	3.0772	3.8374

## Data Availability

The data and the datasets used in this task can be acquired from the corresponding author. Because of industrial confidentiality reason, the data are not publicly available.

## Supplementary Materials

The following are available online at https://www.mdpi.com/article/10.3390/nano11123305/s1, Figure S1: SEM image of AgCl crystal without Ag particles, Figure S2: SEM image of AgCl@Ag crystal with few Ag particles, Figure S3: SEM image of AgCl@Ag crystal with more Ag particles, Figure S4: Original SEM image of AgCl particles (i), zoomed in image of ROI (j), super-resolved image of ROI (k), (l) UV-Vis spectra of AgCl particles as a catalyst to catalyze the degradation of MO.

## Abbreviations

SEM	Scanning Electron Microscopy
RCAN	Residual Channel Attention Network
MAE	Mean Absolute Error
PSNR	Peak Signal-to-Noise Ratio
GAN	Generative Adversarial Network
SSIM	Structural Similarity
RCAB	Residual Channel Attention Block
RIR	Residual In Residual
LSC	Long Skip Connection
SSC	Short Skip Connection
CA	Channel Attention
RG	Residual Group
IFC	Information Fidelity Criterion
