# Supplementary material for: An Adversarial Learning Approach for Super-Resolution Enhancement Based on AgCl@Ag Nanoparticles in Scanning Electron Microscopy Images
Source: Nanomaterials (Basel). 2021 Dec 6;11(12):3305. doi: 10.3390/nano11123305 (PMC8703353; doi:10.3390/nano11123305)
Supplement: Supplementary file 1 [file nanomaterials-11-03305-s001.zip › nanomaterials-1473436-supplementary.pdf]

nanomaterials

MDPI

*Supplementary Materials*

# An Adversarial Learning Approach for Super-Resolution Enhancement Based on AgCl@Ag Nanoparticles in Scanning Electron Microscopy Images

**Li Fan, Zelin Wang, Yuxiang Lu and Jianguang Zhou ***

Research Center for Analytical Instrumentation, Institute of Cyber-Systems and Control, State Key Laboratory of Industrial Control Technology, Zhejiang University, Hangzhou 310027, China; 11732041@zju.edu.cn (L.F.); 12032052@zju.edu.cn (Z.W.); yuxianglu@zju.edu.cn (Y.L.)

* Correspondence: jgzhou@zju.edu.cn

## 1. Synthetic Methods for Nanostructures

The reagents and solvents used in this experiment were purchased from commercial sources and used without further purification. Chemical reagents include nitric acid ($HNO_3$, AR), silver nitrate ($AgNO_3$, AR 99.8%, Sinopharm Chemical Reagent Co., Ltd., China), hydrochloric acid (HCl, AR), ethylene glycol (EG, ≥ 99%) diallyldimethylammonium chloride (DDA) and sodium borohydride ($NaBH_4$, AR, 98%) were purchased from Aladdin Company. All solutions were prepared with ultrapure water (18 M▪cm) and purified by milliq laboratory system (Nihon Millipore Ltd.).

Add 20 mL of ethylene glycol (EG) and 0.8 mL of diallyl dimethyl ammonium chloride (DDA) to a 50 mL flask, stir vigorously for 1 min at room temperature and mix well. Then add 300 μL of 0.1 M $AgNO_3$. Shake for 1 min, then sonicate for 1 minute to mix well. The above solution was placed in an oil bath at 190 °C for reaction. All glassware used in the experiment was immersed in a freshly prepared aqua regia ($HCl:HNO_3 = 3:1$) bath for 30 min and washed with deionized water and ethanol for 3 times. When the reaction was over, the flask was cooled to room temperature, centrifuged at 10,000 r/min for 15 min, the supernatant was removed, washed with ethanol and water twice, and the prepared nanoparticles were collected for use. Use 0.02 M $NaBH_4$ solution for reduction. By adjusting the volume of reducing agent and controlling the ratio of $NaBH_4$ to AgCl sample (R), Ag@AgCl with different degrees of reduction was prepared.

## 2. Catalytic Characterization of Prepared AgCl Crystals

The photocatalytic performance of Ag@AgCl microcrystals was evaluated by using a 300 W xenon arc lamp with a UV cut-off filter as a visible light ($\lambda > 400$nm) source for MO degradation. First, 50 mg of the sample was dispersed in 50 mL MO solution (10 mg/L) and stirred in the dark for 60 min to balance the surface absorption of the catalyst. Then, the mixed solution was exposed to visible light and 3 mL of the solution was taken every 10 min to measure the absorbance.

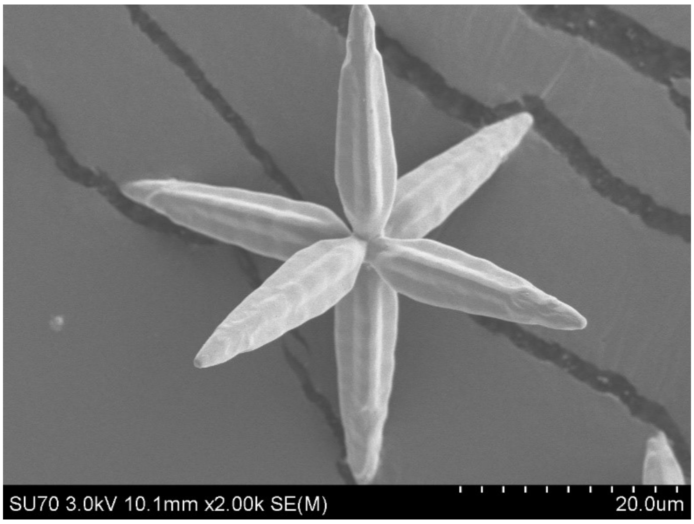


**Figure S1.** SEM image of AgCl crystal without Ag particles.

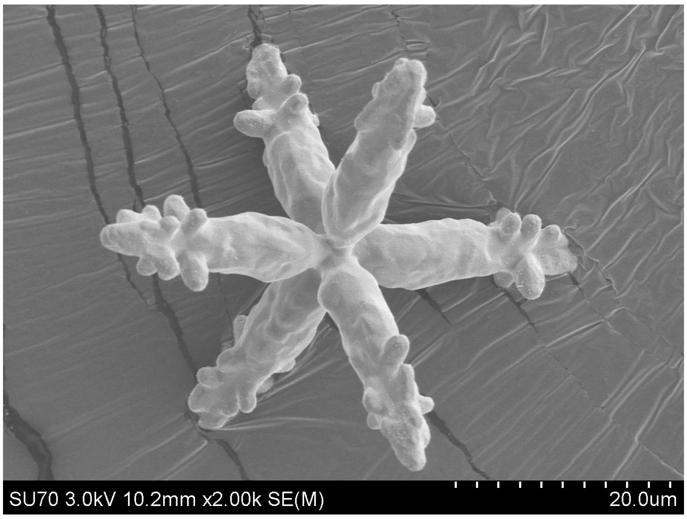


**Figure S2.** SEM image of AgCl@Ag crystal with few Ag particles.

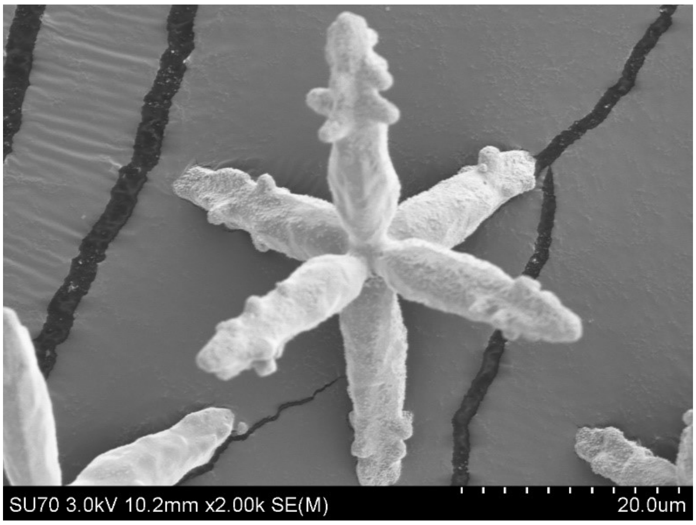


**Figure S3.** SEM image of AgCl@Ag crystal with more Ag particles.

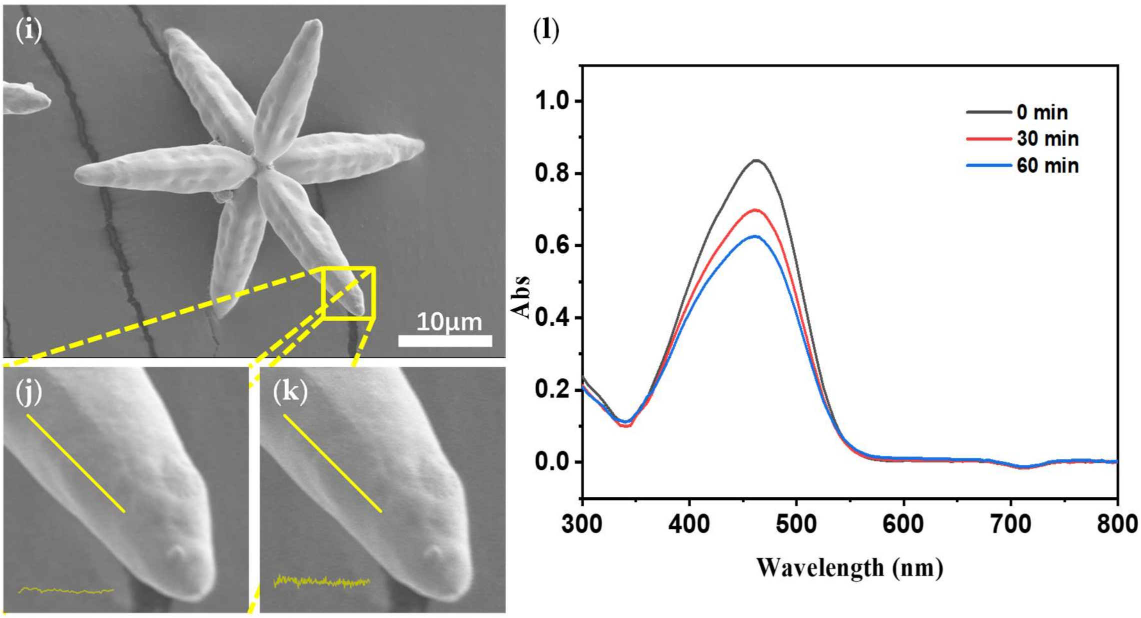


**Figure S4.** Original SEM image of AgCl particles **(i)**, zoomed in image of ROI **(j)**, super-resolved image of ROI **(k)**, **(l)** UV-Vis spectra of AgCl particles as a catalyst to catalyze the degradation of MO.
